# Beyond the Salad: Evaluating the Availability and Healthfulness of Foods Containing Fruits and Vegetables at Convenience Stores

**DOI:** 10.3390/nu18071049

**Published:** 2026-03-25

**Authors:** Claudia J. PromSchmidt, Anna Bernhardt, Nathaniel R. Johnson, Shanon L. Casperson, Derick Thompson, Julie M. Hess

**Affiliations:** 1 Department of Nutrition and Dietetics, University of North Dakota College of Nursing and Professional Disciplines, Grand Forks, ND 58202, USA; 2 USDA-ARS Grand Forks Human Nutrition Research Center, Grand Forks, ND 58203, USA

**Keywords:** food environment, fruits and vegetables, processed foods, convenience stores

## Abstract

**Objective:** The study aimed to evaluate the availability and healthfulness of fruits and vegetables offered at convenience stores in areas of higher and lower levels of food access and household income. **Methods:** A list of products that could count towards servings of fruits or vegetables according to the 2020–2025 Dietary Guidelines for Americans (DGA) was compiled at six convenience store locations, three of which were in low-income (LI) and low-food-access areas (LA). Foods were manually matched with an equivalent item in the Food Patterns Equivalents Database to estimate their fruit or vegetable cup-equivalents. The nutrient density of each product was also determined using criteria from the Robert Wood Johnson Foundation’s Healthy Eating Research (HER) Guidelines. Data collection took place during the summer of 2024 in the Grand Forks, North Dakota, and East Grand Forks, Minnesota Metropolitan Area. **Results:** A total of *n* = 46 products contained at least a one cup-equivalent of fruits and vegetables, and only one product was categorized as one to “choose often” according to HER guidelines. Ten products were found at LILA locations only, 17 were found at non-LILA locations only, and 19 were found at both. Overall, there were no consistent and significant differences in the availability or healthfulness of fruit and vegetable options at convenience stores in LILA compared to non-LILA areas. **Conclusions:** Convenience stores in a small city had few options providing a serving of fruits and vegetables as outlined in DGA recommendations. Shelf-stable fruit and vegetable products may be easier for convenience stores to offer than fresh produce and still contribute to nutrient needs and healthy dietary patterns.

## 1. Introduction

Adequate fruit and vegetable consumption helps prevent chronic disease [1,2,3,4]. Yet, according to the 2020–2025 Dietary Guidelines for Americans (2020–2025 DGA), almost 80% and 90% of the population, respectively, do not meet recommendations for fruit and vegetable intake [5]. Data from the DGA further indicate that Americans eat more grains and protein foods compared to fruits, vegetables, and dairy foods [5]. This disparity in food group consumption can lead to increased risk of obesity, cardiovascular disease, and diabetes, with individuals of low socioeconomic status being particularly affected [6,7,8,9].

There are several barriers to fruit and vegetable intake, especially among lower-income and food-insecure Americans who face challenges, including food access, food security, and food costs [5,10,11,12,13,14]. Processed fruit and vegetable options such as raisins, salsa, and pickled vegetables contribute towards DGA recommendations and may be more likely to be sold at convenience stores than fresh options. However, these foods may also have poor nutritional quality. Increased use of convenience stores is associated with both lower diet quality and food insecurity [15,16,17]. With almost 48 million Americans experiencing food insecurity in 2024 [18,19], more households may be relying on convenience stores for their food purchases [18]. Low-income and low-access (LILA) areas are areas where the poverty rate is 20% or higher, and residents live at least 1 mile from fresh food grocery stores [20]. Residents in these areas are more likely to have difficulty accessing and affording healthy food.

Because convenience stores are often the nearest, most accessible grocery option for those living in LILA areas, it is critical to better understand food choices available at convenience stores and their nutrient profiles [21]. The purpose of this project is to compare access and nutrient-density of fruit and vegetable options at convenience stores both inside and outside LILA areas in the contiguous Greater Grand Forks Metropolitan Area, including both Grand Forks, North Dakota (ND) and East Grand Forks, Minnesota (MN), a community representative of small metropolitan cities in the Upper Midwest and Plains regions of the U.S. The Greater Grand Forks area typifies communities in this region of the U.S., which have limited fresh agri-food market options and access, in part due to extreme winter climates. Grand Forks, for instance, has a farmer’s market in the summer months, but no comparable option in the winter months would be feasible. Extreme weather conditions can also seasonally exacerbate food accessibility concerns in LILA communities, especially among older or disabled adults [22].

We hypothesized that convenience stores in LILA areas will offer more options to meet fruit and vegetable recommendations from the DGA than convenience stores in non-LILA areas with better food access, due to greater demand by those who cannot as easily access grocery stores [16,21]. Robert Wood Johnson Foundation’s Healthy Eating Research (HER) Guidelines were used to evaluate the healthfulness of fruit and vegetable options available at convenience stores in LILA and non-LILA areas.

## 2. Materials and Methods

### 2.1. Regions of Focus

Greater Grand Forks, a small metropolitan area in the United States, offers the opportunity to explore the healthfulness of convenience store offerings in an environment where access to grocery stores is limited. Grand Forks County, where the city of Grand Forks is located, is only one of five recognized metro counties in ND, a state with 53 total counties. Rural counties in ND tend to have poorer food access than metro counties. For those living in non-metro counties, 56.8% have low access to healthy food in ND compared to 45.9% of those residing in metro counties. Despite living in a metro county, 51.9% of residents of Grand Forks County have limited access to healthy foods, meaning limited access to grocery stores [23]. In Grand Forks County, nearly 21% of residents live 16 km (i.e., 10 miles) or more [24], and 51% live more than 1.6 km (i.e., 1 mile) from a fresh food grocery store, according to the most recent data [23,25]. Thus, even within the city of Grand Forks, access to fresh food grocery stores is limited.

**Figure 1:**
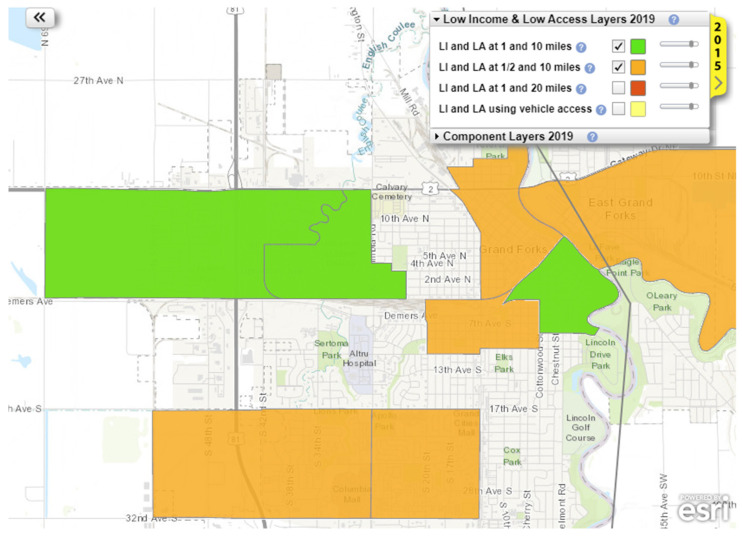
Map of LILA (low-income low-access) and non-LILA areas in Grand Forks [23]. This map is generated the United States Department of Agriculture Economic Research Service. Here is a link to the tool: https://www.ers.usda.gov/data-products/food-access-research-atlas/go-to-the-atlas (accessed on 15 April 2024).

The USDA-ERS offers an online tool (the Food Access Research Atlas) that generates maps of U.S. regions with overlaid indicators of food access and income [23]. Using this tool, a map of Grand Forks, ND, and East Grand Forks, MN was generated with LILA regions at 1.6 and 16 km (1 and 10 miles) and 0.8 and 16 km (½ and 10 miles) highlighted (Figure 1). These LILA regions indicate areas where a significant number of residents are more than 0.5 or 1 mile (urban) or more than 10 miles (rural) from the nearest supermarket.

### 2.2. Convenience Store Identification

As defined by the USDA Supplemental Nutrition Assistance Program (SNAP), a convenience store is “a self-service store that offers a limited line of convenience items and is typically open long hours to provide easy access for customers” [26]. A total of 29 convenience stores were identified in the Grand Forks and East Grand Forks city boundaries using Google Maps^®^ Mountain View, CA, USA; Version 24.47.01.697822364. Convenience stores were divided into LILA and non-LILA locations, using the ERS-generated map (USDA-ERS Food Access Research Atlas; Washington, DC, USA; https://www.ers.usda.gov/data-products/food-access-research-atlas/go-to-the-atlas (accessed on 15 April 2024); Economic Research Service (ERS), U.S. Department of Agriculture (USDA). Food Access Research Atlas, https://www.ers.usda.gov/data-products/food-access-research-atlas/ (accessed on 15 April 2024)) (2024) as a guide [23]. All data from this resource was collected in 2019. Stores were excluded if there was no matching store owned by the same company (e.g., Cenex, Quik Trip, Casey’s) in both LILA and non-LILA areas or if the store did not give permission to collect data (*n* = 14). Stores were matched by company in LILA and non-LILA areas to facilitate equal comparisons. By choosing stores owned by the same company, the only difference between the stores was location (e.g., LILA and non-LILA). Three stores remained that included locations in both LILA and non-LILA from the same company. Three stores each were selected from LILA and non-LILA regions for a total of six convenience stores (i.e., three pairs) visited for this study. LILA and non-LILA locations of the same chain were selected (Table 1), and locations were also selected to include different regions of the metro area.

### 2.3. Convenience Store Product Selection

Once a final list of convenience stores was determined, two members of the research team visited each store together to compile a list of all potential products available at the stores that could count towards servings of fruits or vegetables, as defined by the DGA (Supplemental Table S1). For instance, fruits could include fresh, frozen, canned, and dried options, while vegetables could include fresh, frozen, and canned options. Of products identified as a potential source of fruits or vegetables, a fruit or vegetable must be listed in the top three items on the ingredients list to classify it as a fruit or vegetable. In the U.S., ingredients must be listed on food packages in “descending order of predominance by weight” [27]. Only including foods with fruits and vegetables among the first three ingredients helps to ensure that foods and beverages selected are comprised primarily of fruits and/or vegetables.

Data collected included the names of each specific product, the gram amount of a serving of that product or, when applicable, for the full container, and the price of each product. All data were collected in the summer of 2024.

### 2.4. Data Analysis

#### 2.4.1. Calculating Servings of Fruits and Vegetables from Convenience Store Products

After data collection was completed, the number of servings of fruits and vegetables in each product was calculated. Products were also classified using criteria from the HER guidelines [28] by a separate team member (Supplemental Table S2). First, each food or beverage product was manually matched with an equivalent in the 2017–2018 Food Patterns Equivalent Database (FPED) or Food Pattern Ingredient Database (FPID) (FPED/FPID; Beltsville, MD, USA; Version 2017–2018; https://www.ars.usda.gov/northeast-area/beltsville-md-bhnrc/beltsville-human-nutrition-research-center/food-surveys-research-group/docs/fped-databases/ (accessed on 15 April 2024); Food Surveys Research Group (2017). Food Patterns Equivalents Database (FPED). Food Surveys Research Group Beltsville Human Nutrition Research Center. Dataset. https://hdl.handle.net/10113/AA5880 (accessed on 15 April 2024)) [29]. These databases provide cup-equivalents of fruit, vegetables, and dairy, ounce-equivalents of grains and protein foods, teaspoon-equivalents of added sugars, and gram-equivalents of solid fats and oils per 100 g of each food or beverage product. Matching products to FPED and FPID allowed for the calculation of the servings of fruits, vegetables, solid fats, and added sugars provided by each of the products identified and an estimation of their potential contributions to dietary patterns. Values for each product were calculated by the number of grams in a serving or in a container (when consumption of an entire container is feasible per FDA labeling guidance for dual-column labels [30]). Products were considered to provide some fruits and vegetables if they provided at least 0.1 cup-equivalents of one or more fruit and vegetable variables in FPED or FPID. Using 0.1-cup equivalent as the cutoff enabled the inclusion of foods that may include only small amounts of fruits and vegetables, such as mixed dishes and beverages [31,32]. Previous studies have indicated that mixed dishes are a primary contributor to vegetable intake, and sugar-sweetened and diet beverages are a top contributor to fruit intake [19].

#### 2.4.2. Healthy Eating Research (HER) Guideline Application

Each potential fruit and vegetable product was also evaluated using the 2020 HER Nutrition Guidelines (Healthy Eating Research Program; Durham, NC, USA; https://uconnruddcenter.org/her-guidelines/ (accessed on 15 April 2024); Schwartz, M.; Levi, R.; Lott, M.; Arm, K.; Seligman, H. *Healthy Eating Research Nutrition Guidelines for the Charitable Food System*; 2020.) [28]. The HER guidelines provide a validated framework for evaluating the nutritional value of different foods, specifically in the charitable food setting, where most foods are shelf-stable and processed. This system was developed based on the American food supply and relies on specific product details to classify foods, which is why it was selected for this study. Before selecting HER, two alternate systems developed for convenience stores were also considered, namely, the Short-Form Corner Store Audit Tool (SCAT) [33] and the Nutrition Environment Measures Survey—Corner Store (NEMS-CS) tool [34]. SCAT is a seven-item questionnaire that contains only three questions germane to fruit and vegetable offerings, and it did not capture enough data for this investigation. NEM-CS collects data on the availability, quality, and price of foods, and we piloted the use of this tool before finalizing our research methods. NEM-CS gathers data on all types of healthy and nutrient-poor foods in convenience stores. However, this tool lacks the ability to collect extensive details on fruit and vegetable offerings, specifically an important aspect of our investigation, so combining HER guidelines and FPED/FPID data provided more detailed methods to evaluate fruit and vegetable options.

Products were first placed into one of 11 categories (fruits and vegetables, grains, protein, dairy, non-dairy alternatives, beverages, mixed dishes, processed and packaged snacks, desserts, condiments and cooking staples, and miscellaneous products) [35]. Then, products were grouped into “choose often” (green), “choose sometimes” (yellow), or “choose rarely” (red) based on the content of their nutrients to limit (saturated fat, sodium, and added sugars). The amounts for each nutrient to limit are what determine the placement of foods into different choice groups (often, sometimes, and rarely), and the cut-off points vary by food group (fruits and vegetables, grains, dairy, etc.). For instance, a single serving of a beverage is labeled “choose sometimes” if it has 0 g of saturated fat but “choose rarely” if it has 1 g or more of saturated fat. In contrast, a protein food is labeled “choose sometimes” if it contains 2.5–4.5 g of saturated fat and “choose rarely” if it contains 5 or more grams of saturated fat. All desserts are considered “choose rarely” options, and both “condiments and cooking staples” and “miscellaneous products” are not ranked by choice category (Supplementary Table S2).

#### 2.4.3. Statistical Analysis

Summary statistics were used to compare the proportions of “choose often,” “choose sometimes,” and “choose rarely” foods identified at each store location. HER rankings were also coded into ordinal values (1 = often; 2 = sometimes; 3 = rarely). Data entry and cleaning were conducted in Microsoft Excel and exported to IBM SPSS Statistics for Windows, Version 29 (IBM Corp, Armonk, NY, USA) for analysis. Paired T-tests with the corrected standard deviation of the difference were conducted to compare HER rankings between pairs of LILA and non-LILA stores. A confidence interval of 90% was set to identify significance with a small sample size. Additional models (1) without potato chips and (2) without foods with estimated weights (e.g., trail mixes, berry yogurt parfaits, fresh salads, French fries) were also conducted as sensitivity analyses.

## 3. Results

### 3.1. Products Containing at Least 0.1 Cup-Equivalent of Fruits and Vegetables

The total number of products identified at each convenience store location by the HER food group can be found in Table 2. An average of *n* = 143 items per store provided at least 0.1 cup-equivalent of fruits or vegetables per serving. Items that did not provide at least 0.1 cup-equivalent of fruits or vegetables per serving, such as fruit-flavored drinks, jellies/jams, pizza bites, and dips, were removed from the dataset (*n* = 154). Items that were not independently flagged by both research staffers as potential sources of fruits and vegetables were also removed from the dataset (*n* = 26). A total of *n* = 855 foods and beverages from all six stores combined contained at least 0.1 cup-equivalent of fruit and vegetable servings (Table 2).

### 3.2. Products Containing at Least One Cup-Equivalent of Fruits and Vegetables

Only *n* = 46 products contained at least one cup equivalent, or one serving, of fruits or vegetables (Table 3) [5]. A list of specific items containing at least one cup-equivalent of fruits or vegetables alongside their HER ratings can be found in Supplementary Table S3. Twenty of these products fit into the HER “fruits and vegetables” category, while the others were mixed dishes, beverages, or desserts. Only one food included in the list of one-cup-equivalents of fruits and vegetables, a fresh apple, was described as a food that can be consumed “often,” and the other food items are labeled as “eat sometimes” or “rarely.” Only 10 of these products are available at LILA stores, while 17 of these foods are at non-LILA stores, and 19 products are available at both LILA and non-LILA stores.

### 3.3. Products by HER Choice Category and Comparing LILA and Non-LILA

The foods at each LILA and non-LILA store that fit into the “often,” “sometimes,” or “rarely” categories are presented in Table 4. For some foods, not enough information was available to place them into any one of these three categories. To apply the HER guidelines, a complete Nutrition Facts panel is needed to determine whether to choose “often”, “sometimes”, or “rarely.” One non-LILA store offered fresh fruit, yogurt parfaits, and French fries. The yogurt parfait had some nutrition information provided, but there was not a gram amount of total or added sugars listed, so a determination on its choice categorization could not be made. No nutrition information was listed for the French fries. Other products (*n* = 7) also did not have sufficient nutrition information listed to allow a determination, including packaged mandarin oranges, packaged mixed and tropical fruits, packaged fruits in gelatin, berry yogurt parfait, and fresh salad. Of all products able to be assigned a HER category, 68% fell into the “choose rarely” category, and fewer than 9% fit into the “choose often” category (Table 4). On average, 56% of all fruit and vegetable items identified were potato chips, which provide 0.5 cup-equivalents of “starchy vegetables” per 1 oz serving. Potato chips accounted for 47% of foods in the “choose rarely” category.

No differences in food type or choice category emerged from summary statistics comparing LILA and non-LILA stores. Approximately half of “choose often” (44%), “choose sometimes” (52%), and “choose rarely” (50%) products were available at LILA stores compared to non-LILA stores.

To further investigate differences in the healthfulness of fruit and vegetable options at LILA and non-LILA stores (Table 5), we also conducted paired t-tests of the relative amounts of fruit and vegetable products and the frequency with which those products were labeled as “often,” “sometimes,” or “rarely” at each set of stores. Stores 1 and 3 had significantly different (*p* < 0.001) availability of fruit and vegetable options and average HER scores between the LILA and non-LILA stores, while Store 2 did not have significant differences in either availability or HER scores between its LILA and non-LILA stores. However, there were more fruit and vegetable options at Store 1’s LILA location and Store 3’s non-LILA location. There were also more foods to consume “sometimes” or “rarely” at Store 2’s LILA location, but more foods to consume “sometimes” or “rarely” at Store 1’s non-LILA location. There were no differences in results after sensitivity analyses omitting items with estimated weights and potato chips. While there were some significant differences in the availability of fruit and vegetable offerings and the frequency of foods that can be consumed often, sometimes, or rarely at two of the three stores, the direction of these relationships varied.

## 4. Discussion

While several studies examine the nutrient density of food items at convenience stores, this is the first study, to our knowledge, that examines the nutrient density of fruit and vegetable options specifically at convenience stores, comparing availability at LILA and non-LILA stores.

Most fruit and vegetable items at each store, LILA and non-LILA, were considered “choose rarely” according to HER guidelines. However, the LILA stores did not unilaterally offer more or more nutritious fruit and vegetable offerings, disproving our hypothesis. Store 1 had fewer fruit and vegetable options at the LILA store, whereas Stores 2 and 3 had more (Table 4). In addition, Stores 2 and 3 had higher HER score means, indicating fewer fruit and vegetable options that could be consumed often, at their LILA locations, but Store 1 had a higher HER mean score at its non-LILA location. Stores 1 and 3 had significantly different (*p* < 0.001) average HER scores between the LILA and non-LILA stores, while Store 2 did not have significant differences in HER scores between its LILA and non-LILA stores. While there were some differences in the number and quality of fruit and vegetable options at LILA and non-LILA stores, the differences were not clear between the LILA and non-LILA locations, indicating that some other factor besides the store location in a certain neighborhood was likely driving stocking decisions.

None of the convenience stores had many nutrient-dense options providing servings of fruits and vegetables. Only one item fit into the “choose often” category and provided ≥1 serving of fruit and vegetables. Yet, five of the six convenience stores in this study are SNAP retailers [36], meaning that they are authorized to accept government food assistance benefits, sometimes referred to as “food stamps.” SNAP retailers are required to offer several staple food options for sale, including at least three options from four food groups: vegetables or fruits, dairy products, meat, poultry, or fish items, and breads or cereals [37]. This research demonstrates how few options SNAP recipients may have to choose from in the “vegetable and fruit” group when shopping at convenience stores.

The field of public health has studied the effects of offering fresh produce in convenience stores for several years [38,39,40,41,42], as previous studies have also documented limited healthy options in small food retailers [40]. Evidence to date does not indicate a clear benefit of offering fresh produce in consumer food selection. A four-year observational study examining the impact of a Healthy Food Small Retailer Program in North Carolina on consumer purchases found that a modest increase in healthy options did not change consumer purchases [38]. A similar but shorter-term intervention in the UK also had null results [43]. In contrast, an intervention in a California convenience store did increase customer fruit and vegetable purchases by offering a range of fresh produce items [44]. Additional research is needed to ascertain whether providing more nutrient-dense options, including fresh fruits and vegetables at convenience stores, actually leads to an increase in selection or consumption of more nutrient-dense items [19].

A barrier to conducting these studies is the challenges that stocking products poses for store owners [44]. Retailers typically have to source and stock produce independently, change prices of fresh items frequently, experience stocking challenges due to weather conditions, and handle small shipments for fresh foods, which can be costly [39]. A Healthy Corner Store Pilot Program conducted in Canada reported that retailers found the food system environment “unfriendly” for corner stores to sell fresh produce, and reported being unable to sell fresh produce at an affordable price [39]. These retailers believed that selling fresh fruits and vegetables at an affordable price would increase consumption, but perceived the associated cost and labor to supply them as a major barrier [39]. A 2016 report from the USDA notes several U.S. cities that have developed successful Healthy Corner Store initiatives [45]. This report notes that, in order to be successful, Healthy Corner Store initiatives must demonstrate benefits to the business, provide training to store owners and staff on nutrition and handling of fresh produce, connect owners to the community, and focus initially on small goals [45].

Regardless of the potential benefits of offering fresh fruit and vegetables at convenience stores, these foods are not actually being widely offered, even in higher-income areas. A more feasible solution for convenience stores may be to offer more shelf-stable fruit and vegetable options or products that can be stored safely for longer periods. Both the 2020–2025 DGA and the newly released 2025–2030 DGA recognize that frozen, canned, and dried fruits and vegetables, all of which can be stored longer than fresh options, can be convenient ways to meet recommendations for healthy dietary patterns [5,46]. There may be concerns with frozen, canned, and dried products, and high sodium and high added sugar intakes from products preserved in brine or sugar syrup. However, these products are also sources of potassium low-so-dium and no-added-sugar (or products preserved in fruit juice or water) options are also available. These options are not currently widely available in convenience stores but present a unique opportunity for healthful foods that do not spoil as quickly as fresh produce and may therefore pose fewer challenges for retailers [38,47].

Food insecurity is one reason Americans shop at convenience stores [48]. In a 2017 study of a large Midwestern city, approximately one-quarter of households experiencing food insecurity reported difficulty accessing fresh fruits and vegetables [48]. Almost 48 million Americans experienced food insecurity in 2024 [18,19], and nearly 42 million Americans received SNAP benefits in a given month of that same year [49]. Food access, especially healthy food access, is a multifaceted challenge that encompasses more than simply having adequate funds to purchase food [50]. Food access is affected by access to grocery stores, income and socioeconomic status, race and ethnicity, food outlet (supermarkets and restaurants) density, food cost, store location, store type, availability of healthy options within a given store, perceptions of food options, and quality of available food options [50]. Transportation is also a crucial component of food access, highlighted by the extensive research on food deserts [50,51]. Twenty-one percent of Americans receiving SNAP benefits used someone else’s vehicle to travel to the grocery store, and 13 percent “walk, bike, or take public transit or a shuttle to the store” [52]. Inconsistent access to a vehicle can result in shopping at supermarkets when feasible but doing supplemental shopping at store locations closer to home and easier to access, which often includes convenience stores [48].

While many items in convenience stores were identified as a fruit and vegetable option in this study, 94.6% of the products provided less than a one-cup-equivalent serving of fruits or vegetables. Of the fruit and vegetable products that provided a one-cup equivalent, only one item was identified as “choose often” according to HER guidelines. The findings from this study show a lack of fruit and vegetable options at convenience store locations that can be used to meet DGA recommendations. Convenience store retailers face challenges in stocking fresh produce [39]; however, stocking healthy shelf-stable fruit and vegetable items may help mitigate this challenge.

### Limitations

Due to the heterogeneity of food across stores and a small sample size, summary statistics and paired t-tests were calculated. A small sample size can make it difficult to detect significant differences between groups and lacks precision due to wide confidence intervals. Outliers, random chance, and inconsistent findings impact the statistical interpretation of results with a small sample size. In addition, while selecting stores from the same company in LILA and non-LILA areas facilitated comparison, it may also have introduced selection bias.

An additional challenge with the data analysis of the products available at convenience stores is that there is no specific system that has been developed to evaluate the nutritional value of products at this kind of location. Both the FPED/FPID equivalents and the HER guidelines were developed for other uses. FPED and FPID are used to inform the calculation of Healthy Eating Index (HEI) scores, which use generic food item information. HER guidelines are intended specifically for use in charitable food settings to provide a consistent system for food banks to evaluate the nutritional value of foods they offer [35,53]. NEMS-CS can be used to evaluate the overall food environment in corner stores, but it does not evaluate the nutritional value of individual products [33].

The use of HER and FPED/FPID presented challenges. Firstly, products with no nutrient fact panels (e.g., salads, fruit cups, and yogurt parfaits) could not be assigned to a HER category. Secondly, items such as hash browns, French fries, and dried cranberries with no specified gram amount on the pack had to have their weight estimated, which impacted how many items contributed to one-cup-equivalent and 0.1-cup-equivalent categories. A gram amount was estimated using the nutrition facts panels of similar products, then the product was matched to an FPED/FPID code. Similarly, there were some fresh fruits that did not meet the HEI criteria for a one-cup equivalent due to the gram amount estimated. Using Food Data Central, bananas (offered at both LILA and non-LILA Stores 1 and 2) were estimated as medium bananas, which weigh 113 g [54]. The oranges were estimated to have a 6.6 cm (2.6 inch) diameter, which weighed 141 g. Despite being fresh fruits, these bananas and oranges were too small to provide one cup-equivalent of fruit. The dried cranberries were included in a product that contained other foods as part of a “snack pack.” However, only the weight of the entire package was available. The entire package weight was used to estimate the fruit servings from dried cranberries in the product, an overestimation. Thirdly, there were challenges in determining the food group placement of certain products. The HER guidelines (Supplementary Table S2) list examples of products that may fall under each food group but do not provide extensive details. For example, certain trail mixes could be categorized as protein foods, processed/packaged snacks, or fruits and vegetables. The weight of nuts to dried fruit in the trail mixes was also not specified on the pack, complicating the cup-equivalent determinations. Some of these trail mixes were removed from the dataset due to having less than 0.1 cup-equivalent of fruit. Trail mixes of predominantly dried fruit were classified as a “fruit” according to HER guidelines (Supplementary Table S2). Also, pickles were classified in the “fruit and vegetable” category, and jam was considered a “condiment,” although it was unclear exactly which HER category these items fall under. These estimations could have artificially increased or decreased the mean HER ratings of each store and affected our conclusions.

Additionally, the process of determining which products to include in this analysis may have presented limitations. Two members of the research team conducted all data collection together to ensure alignment on the inclusion or exclusion of different products. All foods had to contain a fruit or vegetable among the first three ingredients. However, some foods and beverages could have been overlooked either by the research team members or because a fruit or vegetable was not among the first three ingredients. Some items, such as potato chips, smoothies, ice cream, and frozen dinners, contained fruit and vegetable servings, while being high in nutrients to limit.

Variance among food preparation and processing also raised questions among the researchers to adequately define what “fits” the DGA definition of a fruit or vegetable. For example, potatoes (a vegetable) are the first ingredient in potato chips. This product is fried, so it does not precisely meet the DGA description of vegetables as either fresh, frozen, or canned [5]. Yet in FPED/FPID, the potato content still “counts” as a starchy vegetable serving. There was also the question of when to consider corn a vegetable or a grain. Botanically, corn is a fruit because it is a part of the plant that flowers [55]. Vegetables are considered leaves, stems, roots, and bulbs of plants, so corn could be considered a vegetable depending on what part of the plant is being consumed [55]. Corn is also rich in starch, which is why it is categorized as a starchy vegetable. Because corn is a grass, it is also technically a grain [55]. The American Diabetes Association classifies corn as a starch given its carbohydrate content [56], but maturity and moisture impact its nutritional value [57]. When corn is fully mature and dry, it is considered a grain, but when corn is harvested before it is fully mature and the kernel is soft, it is considered a vegetable [57]. Certain products like corn chips and corn nuts were taken out of the dataset due to their potential of being classified as a grain according to the USDA [5,58] and the DGA [5]. Although these foods technically provide a serving of fruit or vegetable, potato chips and ice cream are foods that are high in nutrients to limit and not recommended as sources of fruits and vegetables. Including foods like chips in our dataset could have inflated our estimates of the number of vegetables offered at different stores without strict adherence to DGA recommendations.

## 5. Conclusions

Although conducted in a small number of stores in a representative city, few options in the convenience stores assessed met the criteria to be considered a serving of fruits or vegetables as outlined in the 2020 DGA recommendations. Even fewer options suitable for frequent consumption were available, a challenge for consumers wanting to follow a healthy dietary pattern with limited financial and transportation resources. Stocking fresh produce remains a barrier for convenience store owners who often need to source these perishable and seasonal products independently and at great and variable cost. Some localities have successfully provided additional resources to business owners to allow for greater food access in more communities, including LILA areas, and this strategy may be suitable for the Grand Forks area and similar cities.

When offering fresh produce is not a feasible option for all convenience stores, shelf-stable fruit and vegetable options or frozen options can also contribute to nutrient needs and promote health. More research is needed on whether offering a broader range of fruits and vegetables by storage method (frozen, shelf-stable, and fresh) at convenience stores could lead to an increase in consumption of these important food groups.

## Figures and Tables

**Table 1:** Matched convenience stores in the Grand Forks Metropolitan Area used for study visits.

Convenience Store	LILA (Low-Income Low-Access) Location	Non-LILA Location
Store 1	X	X
Store 2	X	X
Store 3	X	X

**Table 2:** Number of products containing at least 0.1 cup-equivalent of fruits and vegetables by food.

Store	Beverages	Condiments/Cooking Staples	Dairy	Dessert	Fruits and Vegetables	Mixed Dishes	Processed/Packaged Snacks	Protein	TOTAL ^1^
LILA Store 1	0	1	5	4	31	8	78	13	140
Non-LILA Store 1	3	1	4	5	40	24	96	12	185
LILA Store 2	3	1	0	0	17	8	89	5	123
Non-LILA Store 2	1	0	0	2	9	6	63	3	84
LILA Store 3	7	1	0	4	28	30	89	5	164
Non-LILA Store 3	8	1	0	6	38	20	82	4	159
TOTALS (% of total products)	22 (2.6)	5 (0.6)	9 (1.1)	21 (2.5)	163 (19.1)	96 (11.2)	497 (58.1)	42 (4.9)	855

^1^ Note that the total number of products in some cases includes the same product available in different-sized containers.

**Table 3:** Convenience store foods with ≥1 cup-equivalent of fruits or vegetables.

HER Food Category	Often	Sometimes	Rarely	TOTALS
Fruits and Vegetables ^1^	1	14	4	**20 ^1^**
Mixed Dishes	0	1	14	**15**
Processed/Packaged Snacks	0	0	5	**5**
Desserts	0	0	3	**3**
Beverages	0	0	3	**3**

^1^ One item is not represented in a HER choice option due to not enough information available.

**Table 4:** Foods by choice category in convenience stores by low-income low-access (LILA) and non-LILA areas ^1^.

Stores ^1^	Choose Often ^2^	Choose Sometimes (% of Products That Are Potato Chips)	Choose Rarely (% of Products That Are Potato Chips)
LILA Store 1	12	45 (33.3%)	80 (70%)
Non-LILA Store 1	18	51 (29.4%)	111 (67.6%)
LILA Store 2	8	26 (50%)	87 (83.9%)
Non-LILA Store 2	7	12 (66.7%)	65 (81.5%)
LILA Store 3	12	30 (36.7%)	120 (65%)
Non-LILA Store 3b	15	30 (43.3%)	109 (64.2%)
**TOTAL (*n*)**	**72**	**194**	**572**
**Percentage of total foods and beverages (%)**	**8.6**	**23.2**	**68.3**

^1^ Products not captured by this categorization schema include miscellaneous foods and condiments, and cooking staples. In this study, those specific foods include ketchup. ^2^ Choice categories include “choose often”, “choose sometimes”, and “choose rarely”. Categorization is determined by the food and beverage content of saturated fat, sodium, and added sugars.

**Table 5:** Paired comparison of nutrient density of fruits and vegetables at low-income low-access (LILA) and non-LILA convenience stores.

Pair	Mean Difference *	Standard Deviation	Two-Sided *p*
Stores 1	0.246	1.206	<0.001
Store 2	0.106	1.476	0.158
Store 3	−0.378	1.326	<0.001
Without Potato Chips Store 1	0.113	0.815	0.04
Without Potato Chips Store 2	0.068	1.629	0.536
Without Potato Chips Store 3	−0.339	1.282	<0.001

* Mean difference in LILA minus non-LILA healthy eating research scores; healthy eating research guidelines scores where 1 = “choose often,” 2 = “choose sometimes,” and 3 = “choose rarely”.

## Data Availability

Data available in a publicly accessible repository upon publication.

## Supplementary Materials

The following supporting information can be downloaded at: https://www.mdpi.com/article/10.3390/nu18071049/s1, Table S1: Items that can serve as servings of fruit or vegetables as specified in the 2020-2025 Dietary Guidelines for Americans; Table S2: Descriptions of each food category in the Healthy Eating Research Guidelines; Table S3: Specific List of Convenience Store Foods With >1 Cup-Equivalent of Fruits or Vegetables.

## Abbreviations

The following abbreviations are used in this manuscript:

DGA	Dietary Guidelines for Americans
ERS	Economic Research Service
FPED	Food Patterns Equivalent Database
FPID	Food Pattern Ingredient Database
HEI	Healthy Eating Index
HER	Healthy Eating Research
LA	Low-Access
LI	Low-Income
MN	Minnesota
ND	North Dakota
NEM-CS	Nutrition Environment Measures Survey—Corner Store
SCAT	Short-Form Corner Store Audit Tool
SNAP	Supplemental Nutrition Assistance Program
