# Supplementary material for: Beyond the Salad: Evaluating the Availability and Healthfulness of Foods Containing Fruits and Vegetables at Convenience Stores
Source: Nutrients. 2026 Mar 25;18(7):1049. doi: 10.3390/nu18071049 (PMC13074773; doi:10.3390/nu18071049)
Supplement: Supplementary file 1 [file nutrients-18-01049-s001.zip › nutrients-4155910-supplementary.pdf]

Supplementary Table S1. Items that can serve as servings of fruit or vegetables as specified in the 2020-2025 Dietary Guidelines for Americans.

Vegetables

Dark-Green Vegetables: All fresh, frozen, and canned dark-green leafy vegetables and broccoli, cooked or raw: for example, amaranth leaves, basil, beet greens, bitter melon leaves, bok choy, broccoli, chamnamul, chrysanthemum leaves, chard, cilantro, collards, cress, dandelion greens, kale, lambsquarters, mustard greens, poke greens, romaine lettuce, spinach, nettles, taro leaves, turnip greens, and watercress.

Red and Orange Vegetables: All fresh, frozen, and canned red and orange vegetables or juice, cooked or raw: for example, calabaza, carrots, red chili peppers, red or orange bell peppers, pimento/pimiento, sweet potatoes, tomatoes, 100% tomato juice, and winter squash such as acorn, butternut, kabocha, and pumpkin.

Beans, Peas, Lentils: All cooked from dry or canned beans, peas, chickpeas, and lentils: for example, black beans, black-eyed peas, bayo beans, brown beans, chickpeas (garbanzo beans), cowpeas, edamame, fava beans, kidney beans, lentils, lima beans, mung beans, navy beans, pigeon peas, pink beans, pinto beans, split peas, soybeans, and white beans. Does not include green beans or green peas.

Starchy Vegetables: All fresh, frozen, and canned starchy vegetables: for example, breadfruit, burdock root, cassava, corn, jicama, lotus root, lima beans, immature or raw (not dried) peas (e.g., cowpeas, black-eyed peas, green peas, pigeon peas), plantains, white potatoes, salsify, tapioca, taro root (dasheen or yautia), water chestnuts, yam, and yucca.

Other Vegetables: All other fresh, frozen, and canned vegetables, cooked or raw: for example, artichoke, asparagus, avocado, bamboo shoots, bean sprouts, beets, bitter melon (bitter gourd, balsam pear), broccoflower, Brussels sprouts, cabbage (green, red, napa, savoy), cactus pads (nopales), cauliflower, celeriac, celery, chayote (mirliton), chives, cucumber, eggplant, fennel bulb, garlic, ginger root, green beans, iceberg lettuce, kohlrabi, leeks, luffa (Chinese okra), mushrooms, okra, onions, peppers (chili and bell types that are not red or orange in color), radicchio, sprouted beans (e.g. sprouted mung beans), radish, rutabaga, seaweed, snow peas, summer squash, tomatillos, turnips, and winter melons.

Fruits

All fresh, frozen, canned, and dried fruits and 100% fruit juices: for example, apples, apricots, Asian pears, bananas, berries (e.g., blackberries, blueberries, cranberries, currants, dewberries, huckleberries, kiwifruit, loganberries, mulberries, raspberries, and strawberries); citrus fruit (e.g., calamondin, grapefruit, kumquats, lemons, limes, mandarin oranges, pomelos, tangerines, and tangelos); cherries, dates, figs, grapes, guava, jackfruit, lychee, mangoes, melons (e.g., cantaloupe, casaba, honeydew, and watermelon); nectarines, papaya, passion fruit, peaches, pears, persimmons, pineapple, plums, pomegranates, prunes, raisins, rhubarb, sapote, soursop, starfruit, and tamarind.

Supplementary Table S2. Descriptions of each food category in the Healthy Eating Research Guidelines.

Food Categories	Example Products
Beverages	Water, soda, coffee, tea, sports drinks, non-100% juice products
Condiments and Cooking Staples	Spices, oil, butter, plantbased spreads, flour, salad dressing, jarred sauces (except tomato sauce), seasoning, salt, sugar
Dairy	Milk, cheese, yogurt
Dessert	Ice cream, frozen yogurt, chocolate, cookies, cakes, pastries, snack cakes, baked goods, cake mixes
Fruits and Vegetables	Fresh, canned, frozen, and dried fruits and vegetables, frozen broccoli with cheese sauce, apple sauce, tomato sauce, 100% juice, 100% fruit popsicle
Mixed Dishes	Frozen meals, soups, stews, macaroni and cheese
Processed/Packaged Snack	Chips (including potato, corn, and other vegetable chips), crackers, granola and other bars, popcorn
Protein	Animal (beef, pork, poultry, sausage, deli meats, hot dogs, eggs) and plant proteins (nuts, seeds, veggie burgers, soy, beans, peanut butter
Grains	Bread, rice, pasta, grains with seasoning mixes
Non-Dairy Alternatives	All plant-based milks, yogurts and cheeses
Miscellaneous Products	Nutritional supplements, baby food

Supplementary Table S3. Specific List of Convenience Store Foods With ≥1 cup-equivalent of Fruits or Vegetables.

Food/Beverage Name	HER (Healthy Eating Research) Food Category	HER Choice Options (choose often, sometimes, rarely)	Available at Low-Income, Low Access (LILA) Stores, non-LILA Stores, or BOTH
**Apples**	Fruits and Vegetables	Often	Both
**Banquet Salisbury Steak meal**	Mixed Dishes	Rarely	LILA
**Barrel O'Fun All Dressed (57 g size)**	Processed/ Packaged Snack	Rarely	LILA
**Barrel O'Fun Barbecue Chips (57 g size)**	Processed/ Packaged Snack	Rarely	Both
**Barrel O'Fun Cheddar and Sour Cream Ripple Chips (57 g size)**	Processed/ Packaged Snack	Rarely	Both
**Barrel O'Fun Original (57 g size)**	Processed/ Packaged Snack	Rarely	Both
**Barrel O'Fun Sour Cream and Onion (57 g size)**	Processed/ Packaged Snack	Rarely	Both
**Bistro Salad Chef with Turkey and Ham**	Mixed Dishes	Rarely	non-LILA
**Bistro Salad Santa Fe Style with Chicken**	Mixed Dishes	Sometimes	non-LILA
**Campbell's Chunky Bowls Beef with Country Vegetables**	Mixed Dishes	Rarely	non-LILA
**Campbell's Chunky Soup Beef with Country Vegetables**	Mixed Dishes	Rarely	non-LILA
**Campbell's Spaghetti O's**	Mixed Dish	Rarely	Both
**Campbell's Tomato Juice**	Fruits and Vegetables	Rarely	LILA
**Chef Boyardee Beefaroni Pasta in Tomato and Meat Sauce**	Mixed Dish	Rarely	non-LILA
**CK Apple Juice**	Fruits and Vegetables	Sometimes	Both
**CK Grape Juice**	Fruits and Vegetables	Sometimes	Both
**CK Orange, Peach, Mango Juice**	Fruits and Vegetables	Sometimes	non-LILA

**CK Pineapple Orange Juice**	Fruits and Vegetables	Sometimes	non-LILA
**Del Monte Cut Green Beans**	Fruits and Vegetables	Sometimes	LILA
**Dinty Moore Beef Stew**	Mixed Dishes	Rarely	Both
**Dole Apple Juice**	Fruit and Vegetables	Sometimes	Both
**Dole Orange Juice**	Fruit and Vegetables	Sometimes	Both
**F’real strawberry banana**	Desserts	Rarely	non-LILA*
**f'real Acai Berry Blast**	Desserts	Rarely	Both
**f'real Mango Smoothie**	Desserts	Rarely	non-LILA*
**Fresh salad**	Fruits and Vegetables	Not enough information available	non-LILA
**Hormel Roast Beef and Mashed Potatoes and Gravy**	Mixed Dishes	Rarely	LILA
**Hungry Man Salsibury Steak meal**	Mixed Dishes	Rarely	LILA
**Jimmy Dean Breakfast Bowl Bacon**	Mixed Dishes	Rarely	LILA
**Lean Cuisine Spaghetti with Meat Sauce**	Mixed Dishes	Rarely	LILA
**Minute Maid Apple Juice**	Fruits and Vegetables	Sometimes	Both
**Minute Maid Orange Juice**	Fruits and Vegetables	Sometimes	Both
**Naked Berry Blast**	Fruits and Vegetables	Sometimes	LILA
**Naked Green Machine**	Fruits and Vegetables	Sometimes	LILA
**Sato's Snacks Dehydrated Mango Slices with Chamoy**	Fruits and Vegetables	Sometimes	Both
**Simply Orange**	Fruits and Vegetables	Sometimes	Both
**Stouffer's Bowl Fulls Fried Chicken and Mashed Potato**	Mixed Dishes	Rarely	non-LILA
**Stouffer's Bowl Fulls Steak and Potatoes**	Mixed Dishes	Rarely	non-LILA
**Tropicana Apple Juice**	Fruits and Vegetables	Sometimes	non-LILA
**V8 Original**	Fruits and Vegetables	Rarely	non-LILA
**V8 Spicy Hot**	Fruits and Vegetables	Rarely	Both

**V8 Splash Fruit Medley**	Beverage	Rarely	non-LILA
**V8 Splash Strawberry Kiwi**	Beverage	Rarely	non-LILA
**V8 Splash Tropical Blend**	Beverage	Rarely	non-LILA
**V8 Vegetable Juice**	Fruit and Vegetables	Rarely	Both
**Van Camp's Beanee Weenee**	Mixed Dishes	Rarely	Both

*Food options found in “both” but not identified by both research team members as a potential source of fruits and vegetables.
